# Deletion of the Pyrophosphate Generating Enzyme ENPP1 Rescues Craniofacial Abnormalities in the TNAP^−/−^ Mouse Model of Hypophosphatasia and Reveals FGF23 as a Marker of Phenotype Severity

**DOI:** 10.3389/fdmed.2022.846962

**Published:** 2022-04-28

**Authors:** Hwa Kyung Nam, Emmanouil Emmanouil, Nan E. Hatch

**Affiliations:** Department of Orthodontics and Pediatric Dentistry, School of Dentistry, University of Michigan, Ann Arbor, MI, United States

**Keywords:** craniofacial abnormalities, hypophosphatasia, pyrophosphate (PP_i_), craniosynostosis, fibroblast growth factor 23 (FGF23), tissue non-specific alkaline phosphatase (TNAP), ENPP1

## Abstract

Hypophosphatasia is a rare heritable metabolic disorder caused by deficient Tissue Non-specific Alkaline Phosphatase (TNAP) enzyme activity. A principal function of TNAP is to hydrolyze the tissue mineralization inhibitor pyrophosphate. ENPP1 (Ectonucleotide Pyrophosphatase/Phosphodiesterase 1) is a primary enzymatic generator of pyrophosphate and prior results showed that elimination of ENPP1 rescued bone hypomineralization of skull, vertebral and long bones to different extents in TNAP null mice. Current TNAP enzyme replacement therapy alleviates skeletal, motor and cognitive defects but does not eliminate craniosynostosis in pediatric hypophosphatasia patients. To further understand mechanisms underlying craniosynostosis development in hypophosphatasia, here we sought to determine if craniofacial abnormalities including craniosynostosis and skull shape defects would be alleviated in TNAP null mice by genetic ablation of ENPP1. Results show that homozygous deletion of ENPP1 significantly diminishes the incidence of craniosynostosis and that skull shape abnormalities are rescued by hemi- or homozygous deletion of ENPP1 in TNAP null mice. Skull and long bone hypomineralization were also alleviated in TNAP^−/−^/ENPP1^−/−^ compared to TNAP^−/−^/ENPP1^+/+^ mice, though loss of ENPP1 in combination with TNAP had different effects than loss of only TNAP on long bone trabeculae. Investigation of a relatively large cohort of mice revealed that the skeletal phenotypes of TNAP null mice were markedly variable. Because FGF23 circulating levels are known to be increased in ENPP1 null mice and because FGF23 influences bone, we measured serum intact FGF23 levels in the TNAP null mice and found that a subset of TNAP^−/−^/ENPP1^+/+^ mice exhibited markedly high serum FGF23. Serum FGF23 levels also correlated to mouse body measurements, the incidence of craniosynostosis, skull shape abnormalities and skull bone density and volume fraction. Together, our results demonstrate that balanced expression of TNAP and ENPP1 enzymes are essential for microstructure and mineralization of both skull and long bones, and for preventing craniosynostosis. The results also show that FGF23 rises in the TNAP^−/−^ model of murine lethal hypophosphatasia. Future studies are required to determine if the rise in FGF23 is a cause, consequence, or marker of disease phenotype severity.

## INTRODUCTION

Tissue Non-specific Alkaline Phosphatase (TNAP, *Alpl*) is a membrane-tethered dephosphorylating enzyme. In humans and mice, TNAP is expressed in bone, growth plate cartilage, teeth, brain, kidney and liver (1–3). While the function(s) of TNAP in liver, kidney and brain are still being established (4–7), numerous prior studies have established that TNAP is an essential promoter of tissue mineralization when co-expressed with type I collagen in mineralized tissues (8–11). The primary known function of TNAP in tissue mineralization is to promote calcium phosphate hydroxyapatite crystal growth in pre-mineralized cartilaginous matrix (osteoid) by decreasing levels of the mineralization inhibitor, inorganic pyrophosphate, and by dephosphorylating osteopontin (12–15).

Ectonucleotide Pyrophosphatase/Phosphodiesterase 1 (ENPP1, *Enpp1*) was first identified as an ectoenzyme that generates inorganic pyrophosphate *via* the hydrolysis of nucleotides and nucleotide sugars (16, 17). Data from both mouse and human studies supports the fact that ENPP1 is essential for control of both hard and soft tissue mineralization. As stated above, pyrophosphate itself inhibits tissue mineralization (18, 19). This is well evidenced by the fact that homozygous ENPP1 deficiency in humans leads to a severe form of vascular calcification (GACI; Generalized Arterial Calcification of Infancy) and that loss of function mutations in ENPP1 leads to spine stiffening (OPLL, Ossification of the Posterior Longitudinal Ligament), both of which are recapitulated in mouse models (20–23). Through as yet unknown mechanisms, ENPP1 deficiency also causes osteomalacia and osteoporosis in both humans and mice (24–28).

Deficient expression and/or activity of TNAP enzyme in humans causes a rare, heritable disorder called hypophosphatasia (HPP). Hypophosphatasia in humans has a broad range of severity that corresponds with timing of onset. Symptoms range from lethal and/or severe with perinatal or infantile onset (29), to modest and milder forms with childhood and adult onset (30, 31). Through a series of preclinical studies conducted on the *Alpl*^−/−^ mouse model of infantile hypophosphatasia (TNAP^−/−^ mice), a mineral-targeted recombinant form of TNAP was developed (32, 33). This recombinant enzyme was initially tested in patients with life threatening disease and was shown to improve respiratory function, skeletal mineralization and survival in patients with severe perinatal and infantile hypophosphatasia (34, 35). More recent results confirm safety and longer-term efficacy of this enzyme replacement therapy. In a 7 year follow up to the original trial, skeletal healing was sustained with additional improvements over time in treatment for respiratory function, weight, height, fine motor, gross motor and cognitive skills (36). While this drug is clearly life rescuing and life changing for severely affected hypophosphatasia patients, symptoms such as muscle weakness and craniosynostosis (the premature fusion of growing cranial bones) are not alleviated with treatment (37).

It is not known why or how craniosynostosis develops in hypophosphatasia, nor why this aspect of the disorder is not diminished upon recombinant enzyme treatment initiated even shortly after birth in humans. We previously showed that craniosynostosis in the form of coronal suture fusion and associated skull shape abnormalities develop in the TNAP^−/−^ mouse model of infantile hypophosphatasia (38). Based upon prior studies showing rescue of skeletal mineralization upon genetic ablation of the inorganic pyrophosphate generating enzyme ENPP1 (13), here we sought to determine if the craniofacial skeletal abnormalities of TNAP^−/−^ mice are alleviated upon genetic ablation of ENPP1. Because the phenotype of TNAP^−/−^ was so variable and because FGF23 was previously shown to be high in ENPP1 null mice, here we also investigated circulating levels of intact FGF23 in the single and double mutant mice. Results demonstrate that normalization of pyrophosphate levels in TNAP^−/−^ mice rescues craniofacial and long bone abnormalities, and that severity of the TNAP^−/−^ phenotype correlates with high serum FGF23 levels in these mice.

## MATERIALS AND METHODS

### Animals

Wild type (TNAP^+/+^/ENPP1^+/+^), TNAP heterozygote (TNAP^+/−^/ENPP1^+/+^) and ENPP1 heterozygote (TNAP^+/+^/ENPP1^+/−^) mice were maintained on a mixed 129/SVJ and C57BL/6 genetic background. TNAP^+/−^/ENPP1^+/+^ and TNAP^+/+^/ENPP1^+/−^ mice were bred to generate wild type, single and double mutant mice. All mice were provided free access to modified laboratory rodent diet containing 325 pyridoxine because TNAP is needed for vitamin B6 metabolism (39). Gross skeletal phenotyping was performed by staining with alizarin red and alcian blue. Genotyping was performed by PCR using DNA samples from tail digests, as previously described (40, 41). Because TNAP^−/−^ mice die at ~3 weeks postnatal, to minimize loss of animals due to premature death, mice were euthanized for phenotyping at day 17. All animal procedures were approved by the Institutional Animal Care and Use Committee (IACUC) of the University of Michigan. Sample numbers for all analyses are provided in Supplementary Figure 1.

### Skull Measurements

Because skull bones of TNAP null mice are present but severely under-mineralized, visualization of some skeletal landmarks on micro-computed tomographic images is not possible. Therefore, previously established skull measurements were taken using digital calipers on whole dissected skulls of day 17 mice (38, 42). To account for differences in skull size between the mouse genotypes, skull measurements were normalized by a total skull length measurement (distance from nasale to opisthion). For size normalized measurements, skull length was measured from nasale to paro, skull width was measured between right and left intersections of the squamosal body to the zygomatic process of the squamous portion of the temporal bone, skull height was measured from pari to the inferior portion of the spheno-occipital synchondrosis, inner canthal distance was measured between right and left intersections of the frontal process of maxilla with frontal and lacrimal bones, nasal bone was measured from nasale to nasion, frontal bone length was measured from nasion to bregma and parietal bone length was measured from bregma to pari. Skull landmarks can be visualized in Supplementary Figure 2. Linear distances were measured three times by one operator and an average per mouse was used for statistical comparison between genotypes. Linear measurements were performed without access to genotypes (blinded).

### Micro Computed Tomography

Whole skulls from day 17 mice were scanned by micro computed tomography (micro-CT) using a Scanco μCT 100 micro-computed tomography system and associated software. Scan settings were 18 μm voxel, 70 kVp, 114 μA, 0.5 mm AL filter, and integration time of 500 ms. A threshold of 1,400 was used based upon calculation of the median auto-threshhold for all wild type skulls. Because juvenile mouse calvarial bones do not yet have well developed cortical and trabecular bone, whole bone analysis (no separation of cortical from trabecular bone) of a previously established frontal bone region of interest (1 mm in length, 1 mm in width, depth equivalent to thickness of bone and position starting at a 0.75 mm distance from sagittal and coronal sutures) was performed (38).

Tibial bones from day 17 mice were scanned at an 18 μm isotropic voxel resolution using the eXplore Locus SP micro-computed tomography imaging system (GE Healthcare Pre-Clinical Imaging, London, ON, Canada) and using Microview version 2.2 software and previously established algorithms (38, 43). Trabecular measurements were made in a region of interest defined as 10% of total bone length from the end of the proximal growth plate. Cortical measurements were made in a region of interest defined as 10% of total bone length from the mid-diaphysis.

### Coronal Suture Fusion Assessment

Synostosis of the coronal suture was identified and assessed on whole dissected fixed skulls of day 17 mice that were alizarin red and alcian blue stained under a dissecting microscope by one experienced individual. Fusion of the suture was confirmed by viewing orthogonal sections across the entire length of the coronal suture, as previously described (38, 42). Craniosynostosis was scored as 0 (normal suture anatomy), 1 (diminished width of suture/greater overlap of frontal and parietal bones) or 2 (suture fusion). Significance between genotypes was established using the Fisher’s exact test. Craniosynostosis assessments were performed without access to genotypes (blinded).

### Primary Cell Isolation and Analysis

Primary osteoprogenitor cells were isolated from 15 day-old mouse cranial bones by serial collagenase digestion. Dissected bones were rinsed, digested with 2 mg/ml collagenase P and 2.5 mg/ml trypsin followed by cell isolation *via* centrifugation. This digestion process was performed three times and cells from only the 3rd digest were used for experimentation as cells of this digest were previously shown to be homogenous (44, 45). Cells were pooled from same genotype littermates for experimentation (*n* = 2–4 mice per genotype per cell collection). For proliferation assays, 2 × 10^4^ cells/well were plated in 24 well plates then cultured for indicated number of days in standard media (custom formulated αMEM without ascorbate plus 10% fetal bovine serum and penicillin/streptomycin). Cells were counted in quadruplicate using a hemocytometer and an average cell count per well was used for comparison between groups. Cell metabolic activity was measured by MTT assay (reduction of (3- (4,5-dimethylthiazol-2-yl)-2,5-diphenyltetrazolium bromide, Sigma) after 5 days of culture in standard media. Ectonucleotide pyrophosphatase/phosphodiesterase enzyme activity was measured by cell culture with the colorimetric substrate, p-nitrophenyl thymidine 5′ monophosphate as previously described (40). Alkaline phosphatase enzyme activity was measured by cell culture with the colorimetric substrate NBT/BCIP (Sigma), as previously described (38). Inorganic pyrophosphate was measured in cells after 5 days of culture in differentiation media (aMEM containing 50 μg/ml ascorbate plus 10% fetal bovine serum and penicillin/streptomycin) followed by gentle rinse then 18 h of culture in phosphate free DMEM using a commercially available bioluminescent assay (Lonza). Inorganic phosphate was measured in cells after 5 days of culture in differentiation media (aMEM containing 50 μg/ml ascorbate plus 10% fetal bovine serum and penicillin/streptomycin) followed by gentle rinse then 18 h of culture in phosphate free DMEM using a commercially available colorimetric kit (Abcam).

### Serum FGF23 Assay and Correlation With Phenotypes

FGF23 levels were measured using a commercially available ELISA (Immunotopics, Quidel) for intact FGF23, using EDTA treated plasma from day 5 or 17 old mice.

Correlation of serum intact FGF23 levels with craniosynostosis was performed by calculating the Spearman’s correlation coefficient (*r*) and associated *p*-value. Correlation of body measurements, skull measurements and skull micro-CT parameters were performed by calculating Pearson correlation coefficient (*r*) with associated coefficient of determination (*R*^2^) and *p*-value.

### Statistical Analyses

Primary outcomes were quantitative measurements of skull shape, incidence of craniosynostosis and bone micro-CT parameters. Secondary outcomes were primary cell assays of proliferation, cell metabolism, ENPP1 and TNAP enzyme activities, and media inorganic pyrophosphate and phosphate levels; in addition to FGF23 serum levels and correlation between FGF23 levels and primary outcome phenotype measurements. Descriptive statistics (mean, standard deviation) for each parameter were calculated for all measurements. Comparisons between groups were made using student’s *t*-tests for normal data and the Mann-Whitney test for non-normal data. Statistical significance was established as *p* < 0.05. Sample numbers for experiments are provided in Supplementary Figure 1.

## RESULTS

### Body Phenotypes

**FIGURE 1 |:**
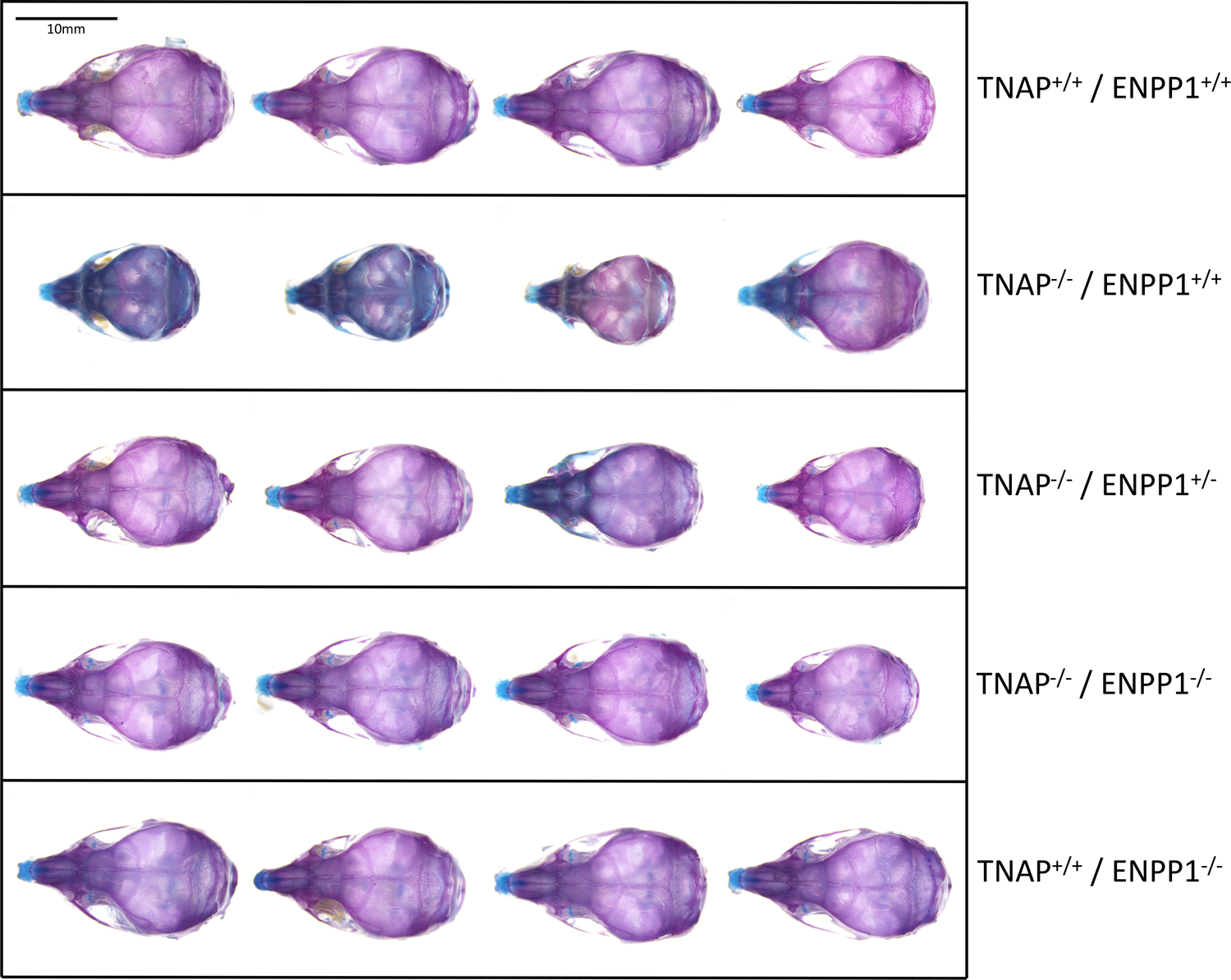
Skull phenotype is rescued by ablation of ENPP1 In TNAP deficient mice. Representative alizarin red and alcian blue stained skulls of each genotype are shown. Note the variable hypomineralization seen in TNAP null (TNAP^−/−^/ENPP1^+/+^) mice.

**FIGURE 2 |:**
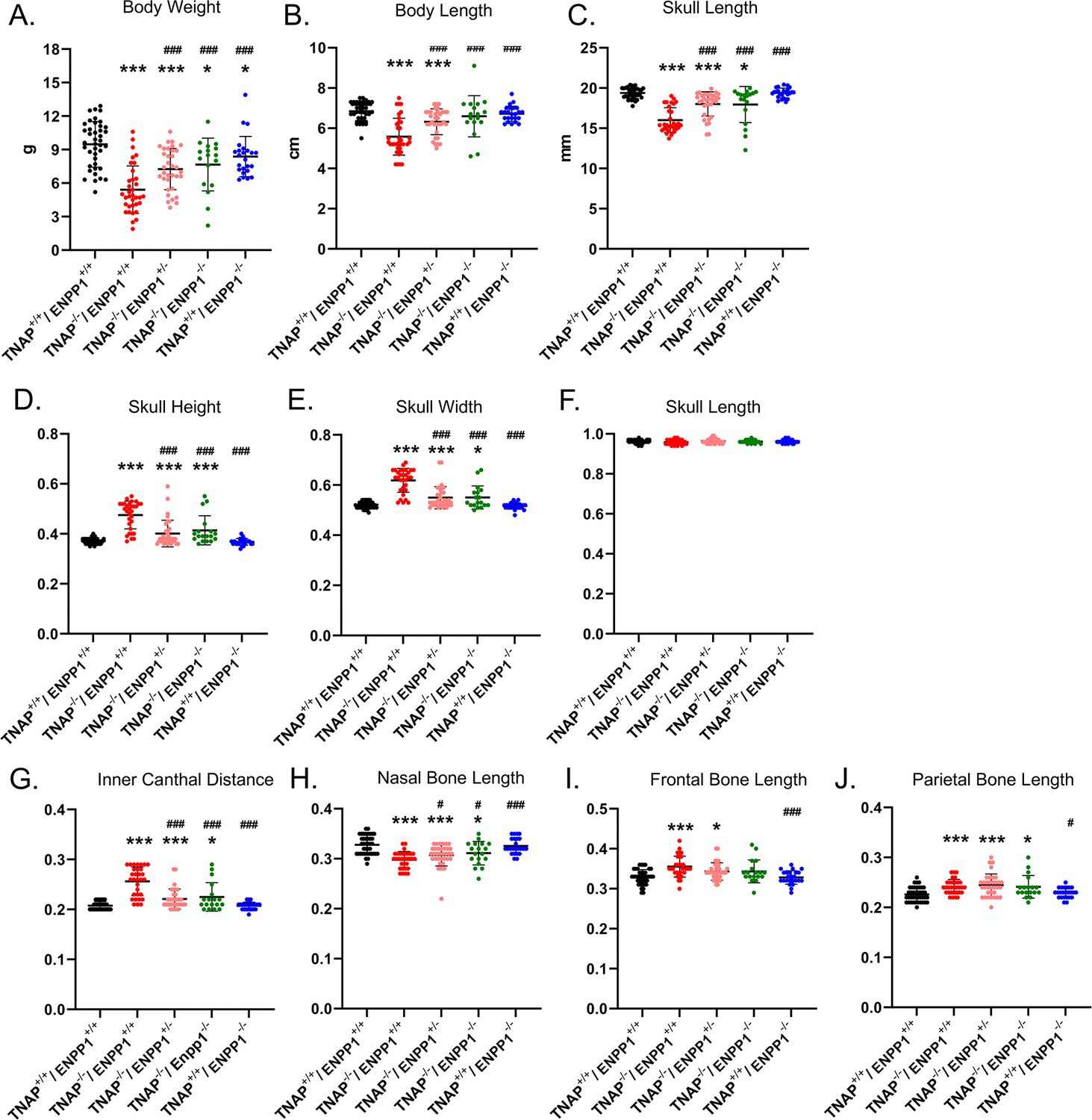
TNAP and ENPP1 ablation effects on body and craniofacial measurements. Total body weight **(A)**, body length **(B)**, and skull length **(C)** for single mutant, double mutant and wild type postnatal day 17 mice are shown. Skull measurements normalized to skull length to account for differences in skull/mouse size for single mutant, double mutant and wild type postnatal day 17 mice are shown **(D–J)**. TNAP^−/−^/ENPP1^+/+^ mice are significantly diminished in body weight, body length and skull size, and exhibit skull shape abnormalities when compared to TNAP^+/+^/ENPP1^+/+^ mice. TNAP^−/−^/ENPP1^−/−^ mice show significantly greater body weight, body length, skull length with improvements in skull shape, when compared to TNAP^−/−^/ENPP1^+/+^ mice. *p < 0.05 vs. TNAP^+/+^/ENPP1^+/+^ mice, ***p < 0.005 vs. TNAP^+/+^/ENPP1^+/+^ mice, ^#^*p* < 0.05 vs. TNAP^−/−^/ENPP1^+/+^ mice, ^###^*p* < 0.005 vs. TNAP^−/−^/ENPP1^+/+^ mice.

Visualization of alizarin red/alcian blue stained skulls revealed an apparent decrease in overall skull size with a shape change of increased skull width plus hypomineralization that was variable in the TNAP null mice (Figure 1). Quantification of phenotypes revealed that all single and double mutant mice were significantly diminished in body weight when compared to wild type mice, with TNAP null mice (TNAP^−/−^/ENPP1^+/+^) weighing significantly less with shorter body and skull lengths than all other genotypes (Figures 2A–C). ENPP1 null mice (TNAP^+/+^/ENPP1^−/−^) were not significantly different in body length or skull size as compared to wild type mice. TNAP/ENPP1 double null mice (TNAP^−/−^/ENPP1^−/−^) were significantly diminished in skull size but not body length when compared to wild type mice. Body length and skull size were also significantly lower in TNAP null (TNAP^−/−^/ENPP1^+/+^) as compared to TNAP/ENPP1 double null (TNAP^−/−^/ENPP1^−/−^), and TNAP null/ENPP1 heterozygous mice (TNAP^−/−^/ENPP1^+/−^).

### Skull Phenotypes

Because overall mouse and skull sizes were different between genotypes (Figures 2B,C), skull linear measurements were normalized by the initial skull length measurement to account for differences in skull size, so as to reveal changes in skull shape not influenced by size. ENPP1 null mice were not different than wild type mice for any skull shape measurement when normalized for skull size (Figures 2D–J). Skull width, skull height and inner canthal distance were all significantly greater in TNAP^−/−^/ENPP1^+/+^ mice as compared to wild type mice, with these TNAP null mice measurements also being significantly greater than those seen in TNAP^−/−^/ENPP1^+/−^ and TNAP^−/−^/ENPP1^−/−^ mice (Figures 2D,E,G). Nasal bone length was significantly smaller in TNAP^−/−^/ENPP1^+/+^ mice as compared to wild type mice, with TNAP null nasal bone length also being significantly smaller than that seen in TNAP^−/−^/ENPP1^+/−^ and TNAP^−/−^/ENPP1^−/−^ mice (Figure 2H). Skull length (nasale to paro), when normalized for skull size, was not different between any of the genotypes (Figure 2F). Frontal bone lengths were greater in TNAP null than wild type mice, and greater than that seen in TNAP^−/−^/ENPP1^+/−^ but not TNAP^−/−^/ENPP1^−/−^ mice (Figure 2I). Parietal bone lengths were greater in TNAP null than wild type mice, and similar to that seen in TNAP^−/−^/ENPP1^+/−^ and TNAP^−/−^/ENPP1^−/−^ mice (Figure 2J).

### Cranial Bone Micro-CT Parameters

**FIGURE 3 |:**
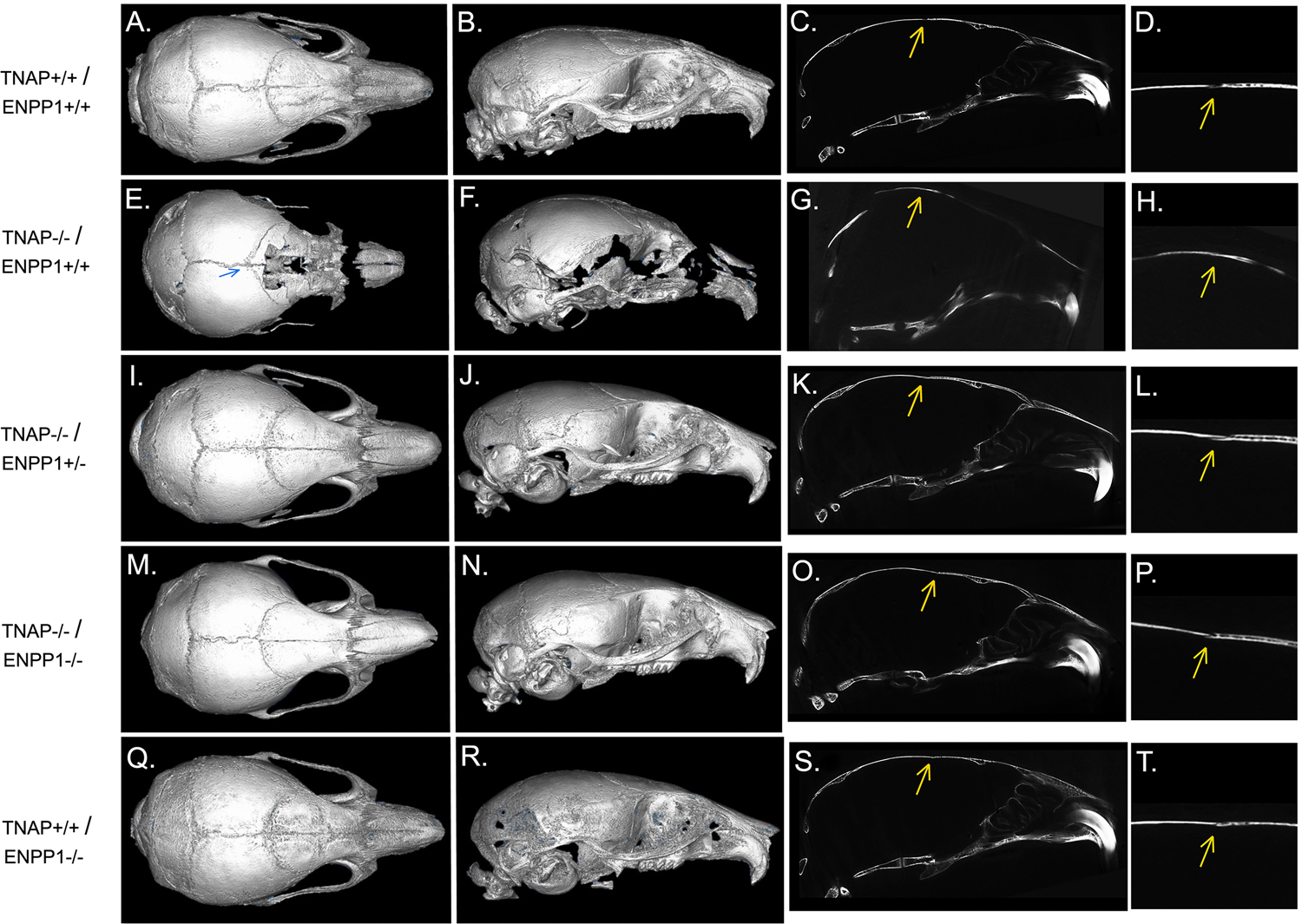
Micro CT Isosurface Images of TNAP and/or ENPP1 mutant mouse skulls. Isosurface and 2D sagittal slice images including magnified image of coronal suture from micro-CT files of TNAP^+/+^/ENPP1^+/+^ mice **(A–D)**, TNAP^−/−^/ENPP1^+/+^ mice **(E–H)**, TNAP^−/−^/ENPP1^+/−^ mice **(I–L)**, TNAP^−/−^/ENPP1^−/−^ mice **(M–P)** and TNAP^+/+^/ENPP1^−/−^ mice **(Q–T)** are shown. Blue arrow indicates site of coronal suture fusion seen on isosurface image of TNAP^−/−^/ENPP1^+/+^ mouse. Yellow arrows indicate site of coronal suture with/without coronal suture fusion in 2D slice images of the mice. Fusion of the coronal suture is only seen in TNAP^−/−^/ENPP1^+/+^ mice **(G,H)**. Also note severe hypomineralization of cranial and facial bones in the severely affected TNAP null mouse. Double null mice appear more similar to wild type mice than the TNAP null mice.

**FIGURE 4 |:**
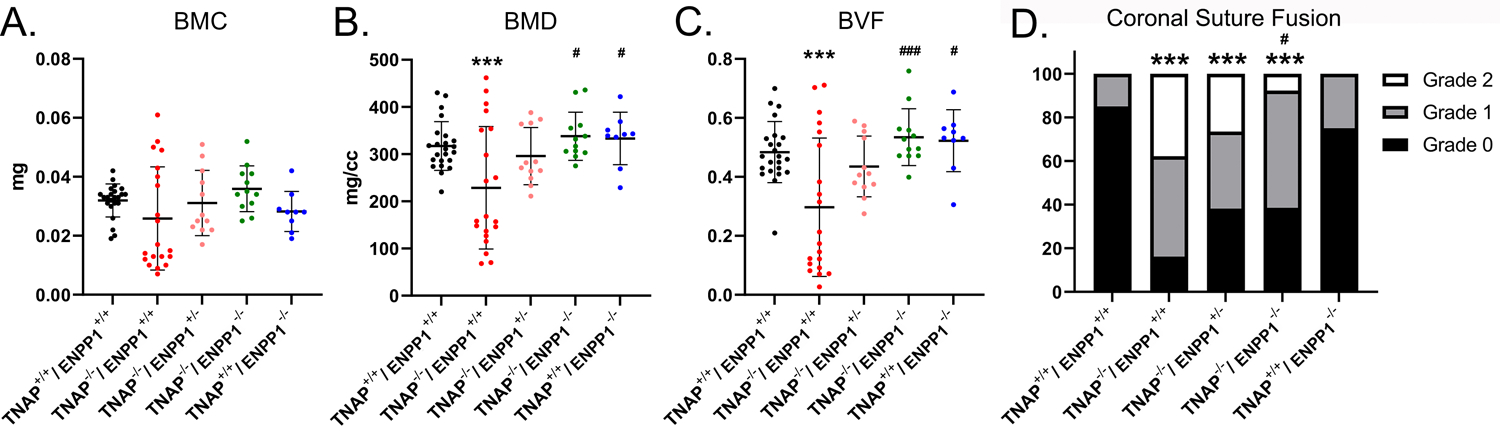
TNAP and ENPP1 ablation effects on cranial bone mineralization and coronal suture synostosis. Bone mineral content (BMC), bone mineral density (BMD) and bone volume fraction (BVF) of the frontal cranial bone is shown **(A–C)**. TNAP^−/−^/ENPP1^+/+^ mice exhibit significantly diminished BMD and BVF when compared to TNAP^+/+^/ENPP1^+/+^ mice. TNAP^−/−^/ENPP1^−/−^ mice show significantly greater BMD and BVF than TNAP^−/−^/ENPP1^+/+^ mice, and are similar to values seen in TNAP^+/+^/ENPP1^+/+^ mice. **(D)** Coronal suture status was scored as 0 (normal suture anatomy), 1 (diminished width of suture/greater overlap of frontal and parietal bones) or 2 (suture fusion). TNAP^−/−^/ENPP1^+/+^, TNAP^−/−^/ENPP1^+/−^, and TNAP^−/−^/ENPP1^−/−^ mice all exhibit some incidence of coronal suture fusion while no coronal suture fusion is seen in TNAP^+/+^/ENPP1^+/+^ mice. TNAP^−/−^/ENPP1^−/−^ mice exhibit a significantly diminished incidence of coronal suture fusion when compared to TNAP^−/−^/ENPP1^+/+^ mice. ***p < 0.005 vs. TNAP^+/+^/ENPP1^+/+^ mice, ^#^*p* < 0.05 vs. TNAP^−/−^/ENPP1^+/+^ mice, ^###^*p* < 0.005 vs. TNAP^−/−^/ENPP1^+/+^ mice.

Isosurface images and 2D sagittal slice images from micro-CT files reveal obvious bone hypomineralization of cranial and facial bones in the TNAP null mice, to the extent that parts of these bones do not appear on micro-CTscans of these mice (Figure 3). Fusion of the coronal suture is seen only in TNAP^−/−^/ENPP1^+/+^ mice (Figures 3G,H). Quantification of micro-CT parameters showed that the frontal cranial bones of TNAP^−/−^/ENPP1^+/+^ mice had significantly diminished bone mineral density (BMD) and bone volume fraction (BVF) but not bone mineral content when compared to wild type frontal bones, and when compared to the frontal bones of TNAP^−/−^/ENPP1^−/−^ mice (Figures 4A–C). Frontal bones of TNAP^+/+^/ENPP1^−/−^ mice showed no significant differences when compared to those of wild type mice. High variability in cranial BMC, BMD, and BVF is evident in cranial bones of the TNAP null mice, as compared to the other genotypes.

### Craniosynostosis

Fusion of the coronal suture was evident in almost 40% of TNAP^−/−^/ENPP1^+/+^ mice, ~30% of TNAP^−/−^/ENPP1^+/−^ mice, and <10% of TNAP^−/−^/ENPP1^−/−^ mice at day 17 (Figure 4D). The incidence of craniosynostosis was significantly higher in TNAP^−/−^/ENPP1^+/+^ mice than in TNAP^−/−^/ENPP1^−/−^ mice, but not when compared to TNAP^−/−^/ENPP1^+/−^ mice.

### Long Bone Micro-CT Parameters

**FIGURE 5 |:**
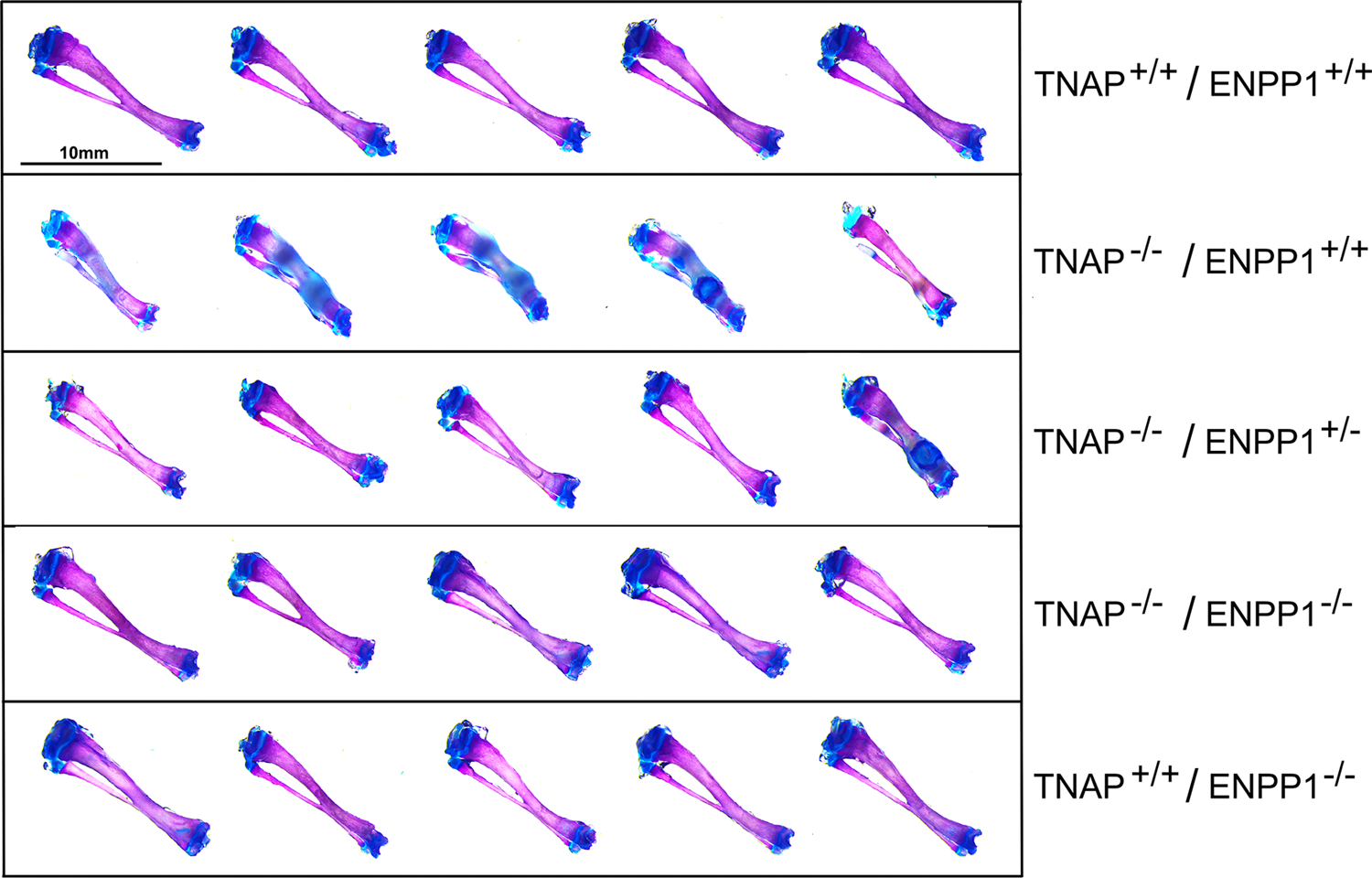
Long bone phenotype is rescued by ablation of ENPP in TNAP deficient mice. Tibias stained with alizarin red and alcian blue of 17-day mice are shown. Severe hypomineralization is variably seen in the TNAP null mice. Double null mice appear more similar to wild type mice than TNAP null mice.

**FIGURE 6 |:**
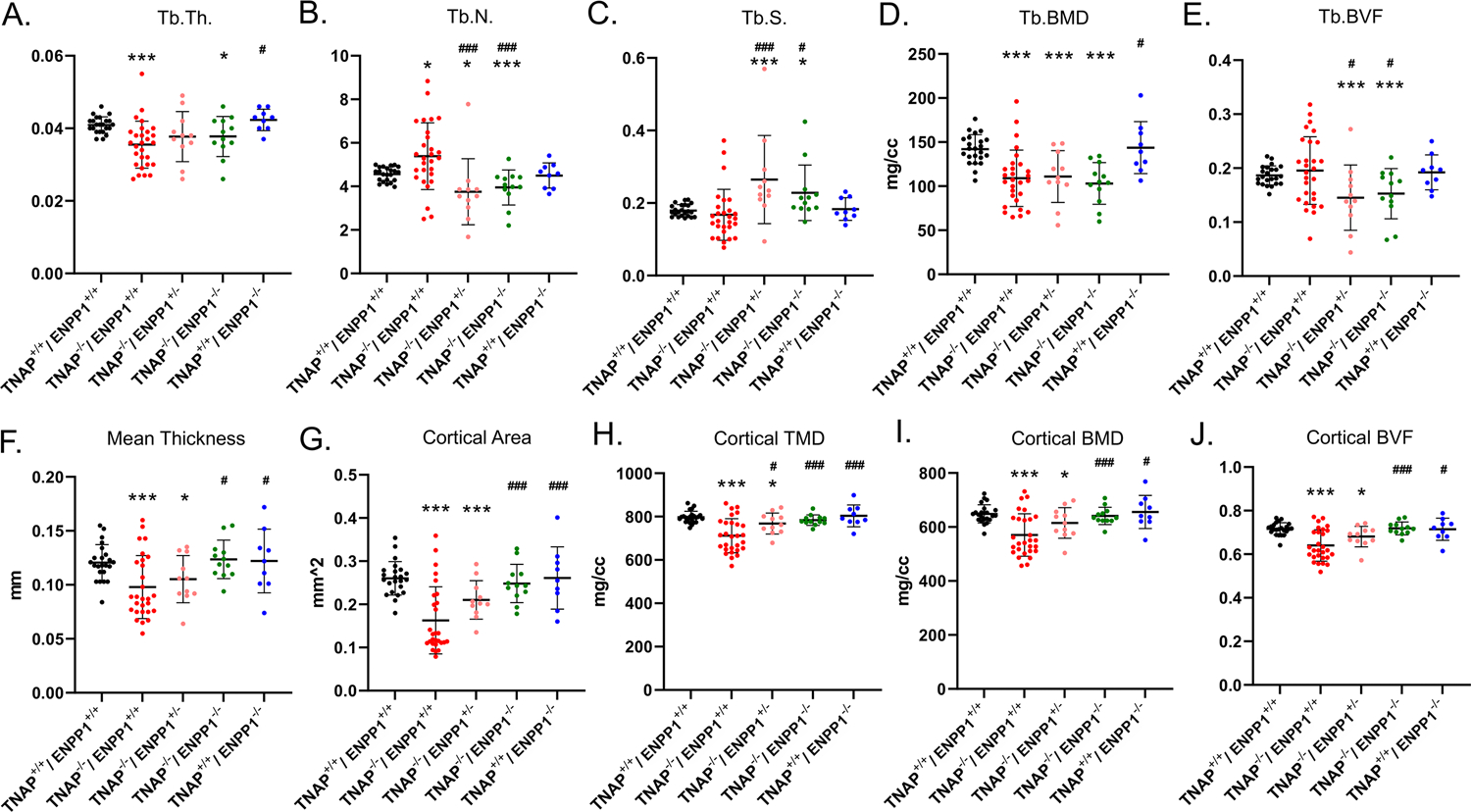
TNAP and ENPP1 ablation effects on long bone. Trabecular thickness (Tb.Th.), number (Tb.N.), spacing (Tb.S.), bone mineral density (BMD) and bone volume fraction (BVF) are shown **(A–E)**. Cortical mean thickness, area, tissue mineral density (TMD), BMD and BVF are shown **(F–J)**. TNAP^−/−^/ENPP1^+/+^ mice are diminished in trabecular thickness and mineral density with increased trabecular number and no difference in bone volume fraction as compared to TNAP^+/+^/ENPP1^+/+^ mice. TNAP^−/−^/ENPP1^−/−^ mice are diminished in trabecular thickness, number and bone volume fraction and have increased trabecular spacing compared to TNAP^+/+^/ENPP1^+/+^ mice. No differences are seen between TNAP^+/+^/ENPP1^−/−^ mice and TNAP^+/+^/ENPP1^+/+^ mice. *p < 0.05 vs. TNAP^+/+^/ENPP1^+/+^ mice, ***p < 0.005 vs. TNAP^+/+^/ENPP1^+/+^ mice, ^#^*p* < 0.05 vs. TNAP^−/−^/ENPP1^+/+^ mice, ^###^*p* < 0.005 vs. TNAP^−/−^/ENPP1^+/+^ mice.

Visualization of alizarin red/alcian blue stained tibias with fibulas revealed hypomineralization to various extents in the TNAP null mice (Figure 5). Quantification of micro-CT parameters (Figure 6) showed that tibial trabecular thickness (Tb.Th.) and trabecular BMD (Tb.BMD) were significantly diminished in TNAP^−/−^/ENPP1^+/+^ compared to wild type mice, and was not rescued (increased) by ablation of ENPP1 in the TNAP null background. Trabecular number (Tb.N.) was increased in TNAP^−/−^/ENPP1^+/+^, and decreased in TNAP^−/−^/ENPP1^+/−^ and in TNAP^−/−^/ENPP1^−/−^ mice when compared to wild type mice. Trabecular spacing (Tb.S.) was increased in TNAP^−/−^/ENPP1^+/−^ and in TNAP^−/−^/ENPP1^−/−^ mice when compared to TNAP null or wild type mice. Trabecular bone volume fraction (Tb.BVF) was diminished only in TNAP^−/−^/ENPP1^+/−^ and in TNAP^−/−^/ENPP1^−/−^ mice. Cortical mean thickness, cortical area, cortical tissue mineral density (TMD), cortical bone mineral density (BMD) and cortical BVF were all significantly diminished in TNAP^−/−^/ENPP1^+/+^ and TNAP^−/−^/ENPP1^+/−^ mice when compared to wild type mice and TNAP^−/−^/ENPP1^−/−^ mice.

### Primary Cell Assays

**FIGURE 7 |:**
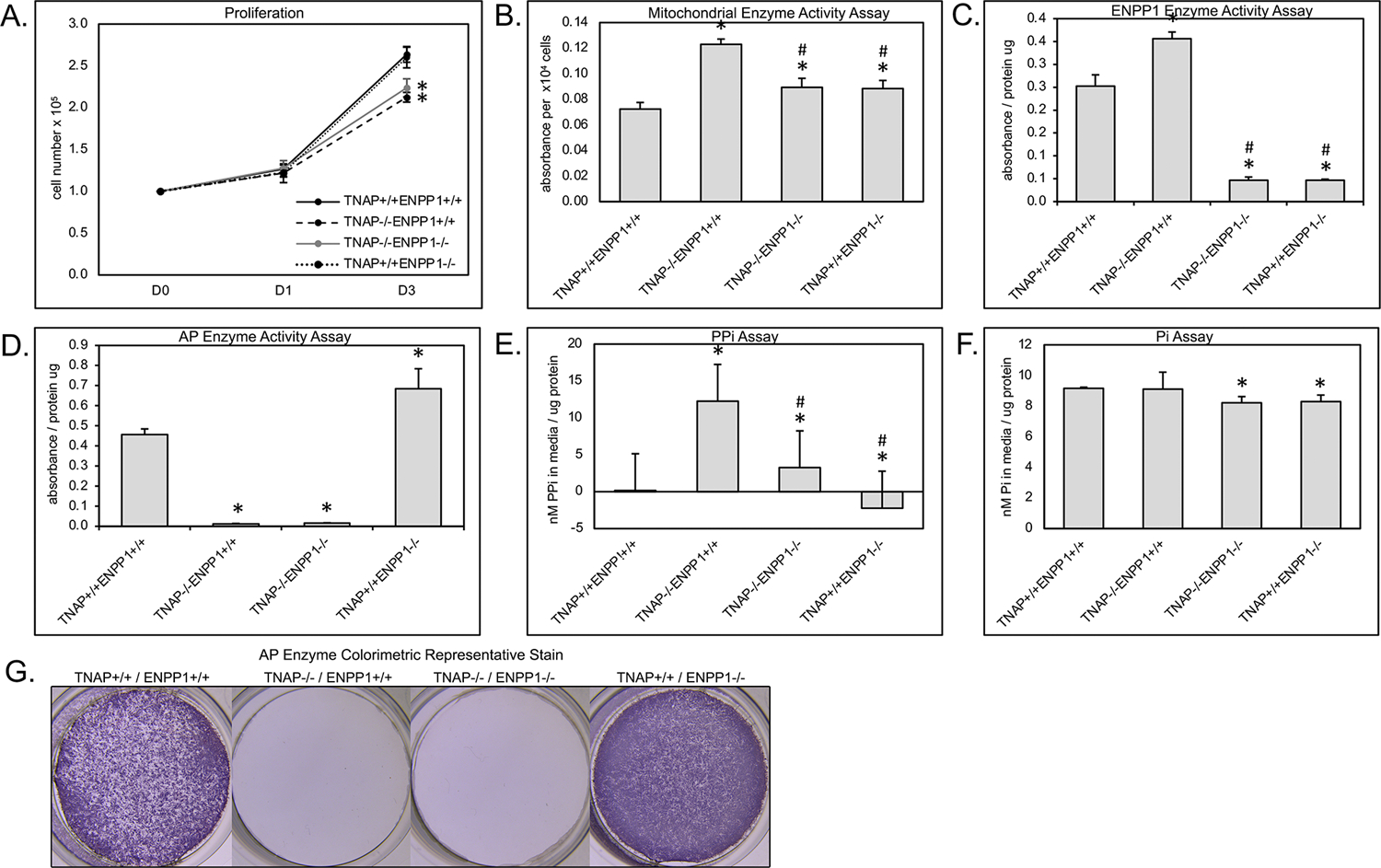
Primary cranial cell proliferation, cell metabolic activity, phosphate and pyrophosphate production. Cell were plated then counted at indicated time points to quantify cell proliferation **(A)**. Colorimetric MTT assay was performed on cultured cells to quantify cell metabolic activity **(B)**. A colorimetric NTPPPH assay was performed on cultured cells to quantify ENPP1 enzyme activity **(C)**. A colorimetric alkaline phosphatase assay was performed on cultured cells to quantify TNAP enzyme activity **(D)**. Visualization of alkaline phosphatase stain **(G)**. Colorimetric assays for inorganic pyrophosphate (PP_i_) **(E)** and phosphate (Pi) **(F)** were performed on media from cultured cells. Both TNAP^−/−^/ENPP1^+/+^ and TNAP^−/−^/ENPP1^−/−^ cells show diminished proliferation compared to TNAP^+/+^/ENPP1^+/+^ and TNAP^+/+^/ENPP1^−/−^ cells. Mitochondrial enzyme activity is increased in TNAP^+/+^/ENPP1^−/−^ and TNAP^−/−^/ENPP1^−/−^ cells, and even more so in TNAP^−/−^/ENPP1^+/+^ cells. ENPP1 enzyme activity is significantly diminished in all cell genotypes in which ENPP1 has been ablated. AP enzyme activity is significantly diminished in all cell genotypes in which TNAP has been ablated. Inorganic pyrophosphate levels are significantly increased in the media of cultured TNAP^−/−^/ENPP1^+/+^ cells. Inorganic phosphate levels are slightly diminished in the media of cells in which ENPP1 has been ablated. *p < 0.05 vs. TNAP^+/+^/ENPP1^+/+^ cells, ^#^*p* < 0.05 vs. TNAP^−/−^/ENPP1^+/+^ cells.

Because we previously found diminished proliferation and increased cellular metabolic activity in primary cells of TNAP null mice (38, 41, 46), we next sought to determine if ablation of ENPP1 in these cells would rescue or worsen these abnormalities. Results show that cells isolated from both TNAP^−/−^/ENPP1^+/+^ and TNAP^−/−^/ENPP1^−/−^ mice exhibit significantly less proliferation when compared to cells from wild type and TNAP^+/+^/ENPP1^−/−^ mice (Figure 7A). Cell metabolic activity as measured by MTT assay was increased in TNAP^−/−^/ENPP1^+/+^ cells when compared to all other genotypes (Figure 7B). Cell metabolic activity was also higher in TNAP^−/−^/ENPP1^−/−^ and TNAP^+/+^/ENPP1^−/−^ when compared to wild type cells, albeit to a lower extent than that seen in the TNAP^−/−^/ENPP1^+/+^ cells.

Cells isolated from all TNAP null genotypes showed minimal TNAP enzyme activity (Figures 7D,G). Cells isolated from ENPP1 single null mice exhibited higher TNAP enzyme activity levels than wild type mice. Cells isolated from all ENPP1 null genotypes showed minimal ENPP1 enzyme activity (Figure 7C). Cells isolated from TNAP single null mice exhibited higher ENPP1 enzyme activity levels than wild type mice.

Measurements of inorganic pyrophosphate (PP_i_) in the media of cultured cells revealed very high PP_i_ levels in the media of TNAP^−/−^/ENPP1^+/+^ cells, which was diminished in TNAP^−/−^/ENPP1^−/−^ cells, but not to the extent of that seen in TNAP^+/+^/ENPP1^−/−^ or wild type cells (Figure 7E). Measurements of inorganic phosphate (P_i_) in the media of culture cells revealed mild significantly diminished P_i_ levels in the media of TNAP^−/−^/ENPP1^−/−^ and TNAP^+/+^/ENPP1^−/−^ cells (Figure 7F). No P_i_ differences were seen in the media of TNAP^−/−^/ENPP1^+/+^ cells.

### Serum FGF23 Levels

**FIGURE 8 |:**
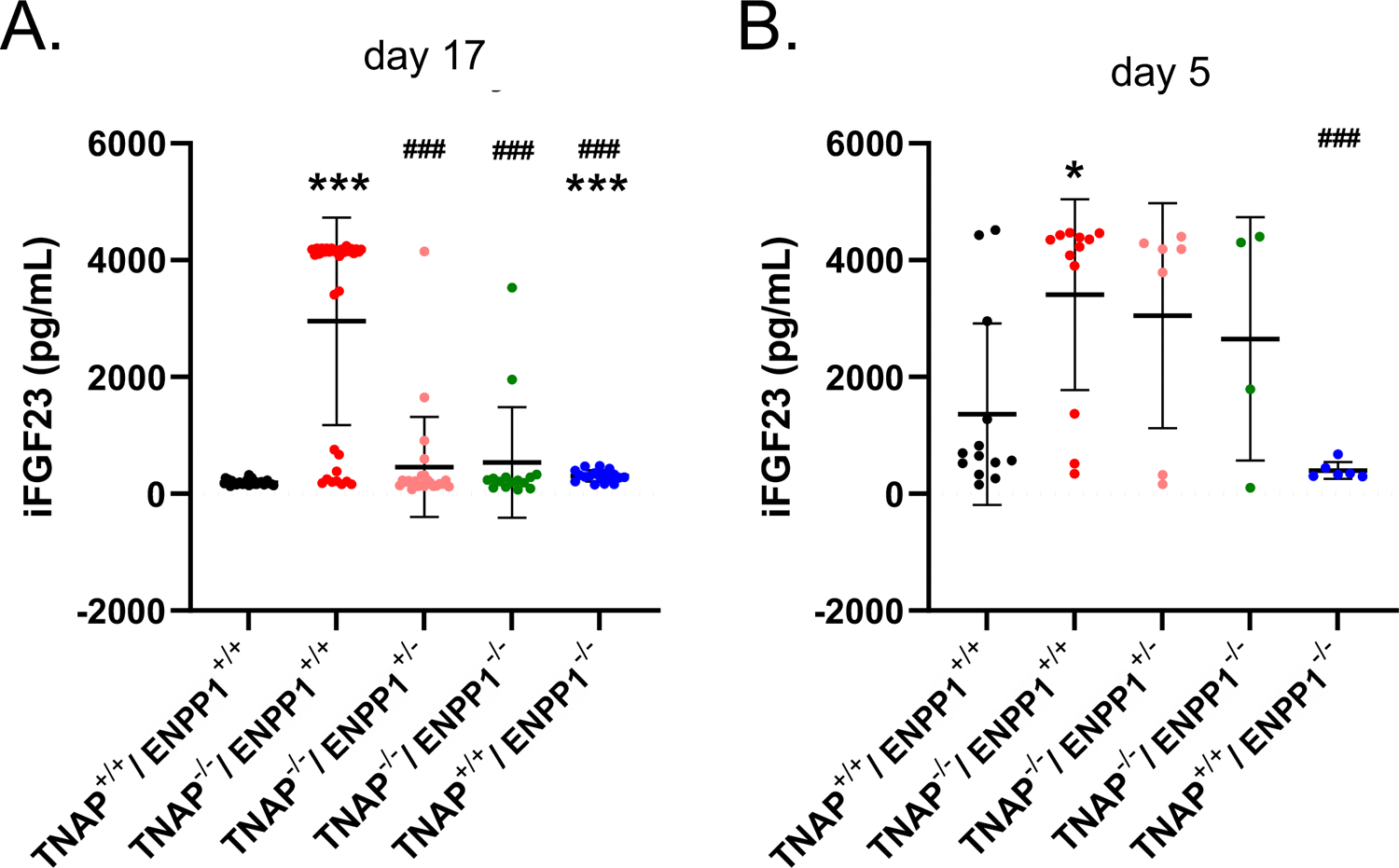
Serum FGF23 levels in TNAP and ENPP1 deficient mice. Serum was isolated from postnatal day 17 **(A)** and postnatal day 5 **(B)** mice and assayed for intact FGF23 by ELISA. Food was withdrawn for 18 h prior to day 17 serum isolation. No food was withdrawn for day 5 serum isolation. Results show increased FGF23 in the serum of TNAP^+/+^/ENPP1^−/−^ mice compared to TNAP^+/+^/ENPP1^+/+^ mice, but even greater levels are seen in the serum of TNAP^−/−^/ENPP1^+/+^ mice at day 17. At day 5, FGF23 levels are more variable in serum but TNAP^−/−^/ENPP1^+/+^ mice still show significantly greater FGF23 than serum from the serum of TNAP^+/+^/ENPP1^+/+^ mice. *p < 0.05 vs. TNAP^+/+^ ENPP1^+/+^ mice, ***p < 0.005 vs. TNAP^+/+^/ENPP1^+/+^ mice, ^###^*p* < 0.005 vs. TNAP^−/−^/ENPP1^+/+^ mice.

Because mice and humans with ENPP1 deficiency exhibit high circulating FGF23 (26, 27) and because FGF23 can suppress TNAP expression (47, 48), we sought to determine if ablation of TNAP in the ENPP1 null background would alter circulating levels of FGF23. While we did find high intact serum FGF23 levels in TNAP^+/+^/ENPP1^−/−^ compared to wild type mice, we found even higher levels in a subset of the TNAP^−/−^/ENPP1^+/+^ mice at day 17 postnatal (Figure 8A). We also tested serum FGF23 levels in younger mice, because day 17 TNAP null mice already have a severe hypophosphatasia phenotype. While more variable than that seen at day 17 due to the inability to perform food withdrawal, intact serum FGF23 levels were also significantly higher in TNAP^−/−^/ENPP1^+/+^ than in wild type mice at postnatal day 5 (Figure 8B).

### Correlation of Serum FGF23 Levels and Phenotypes

**FIGURE 9 |:**
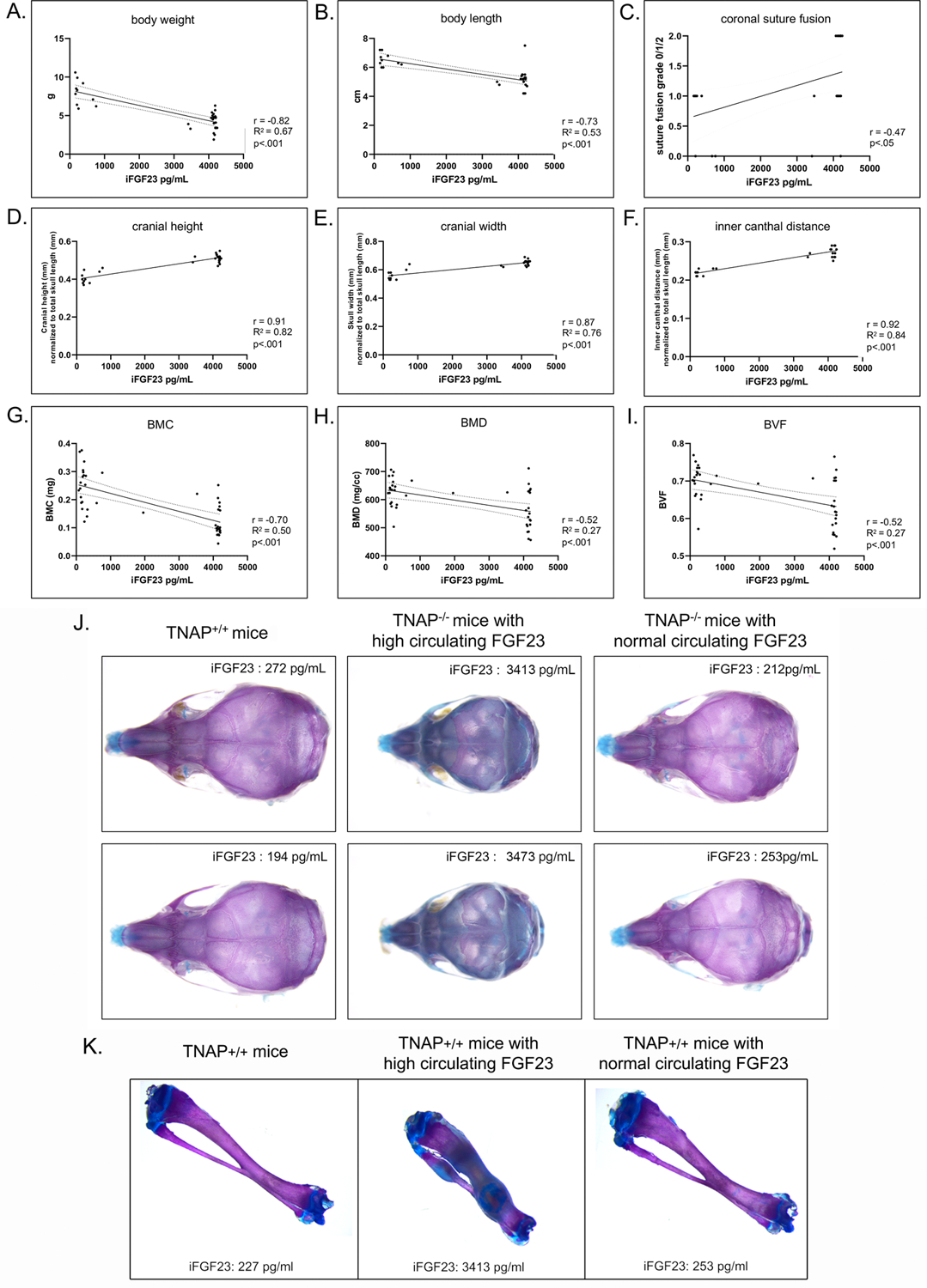
Correlation of FGF23 serum levels with body and skull phenotypes. Body weight **(A)**, body length **(B)**, skull shape measurements **(D–F)** and cranial bone micro-CT measurements **(G–I)** were compared to serum FGF23 levels in TNAP^−/−^ENPP1^+/+^ mice using Pearson correlation coefficient (*r*) with coefficients of determination that indicate validity of the Pearson correlation model (*R*^2^). Coronal suture status score **(C)** (as described for Figure 4) was compared to serum FGF23 levels in TNAP^−/−^/ENPP1^+/+^ mice using Spearman correlation coefficient (*r*). High serum FGF23 levels correlate significantly with decreased body weight, decreased body length, decreased cranial bone mineralization, increased coronal suture fusion, increased cranial height, increased cranial width and decreased inner canthal distance in TNAP^−/−^/ENPP1^+/+^ mice. Representative alcian blue/alizarin red stained skulls are shown **(J)**. TNAP null skulls with normal circulating FGF23 levels look similar to TNAP^+/+^/ENPP1^+/+^ skulls though smaller in size. TNAP null skulls with high circulating FGF23 levels appear shorter in length, thicker in width and have obvious mineralization abnormalities. Representative alcian blue/alizarin red stained tibias are shown **(K)**. Tibias from TNAP null mice with normal circulating FGF23 levels look similar to TNAP^+/+^/ENPP1^+/+^ tibias. Tibias from TNAP null mice with high circulating FGF23 levels are shorter, thicker, hypomineralized and show ectopic cartilage formation.

We next looked for potential correlations between serum FGF levels and phenotypes seen in the TNAP null mice. Correlation of high FGF23 serum levels with skull (Figure 9J) and long bone (Figure 9K) phenotypes appeared to be consistent. Statistical analyses (Figures 9A–I) revealed strong negative Pearson correlations with moderately strong correlations of determination between serum FGF23 levels and body weight (*r* = −0.82, *R*^2^ = 0.67), and between serum FGF23 and body length (*r* = −0.73, *R*^2^ = 0.53). Moderately negative correlations with moderate to low coefficients of determination were found between serum FGF23 levels and cranial bone BMC (*r* = −0.70, *R*^2^ = 0.50), BMD (*r* = 0.52, *R*^2^ = 0.27) and BVF (*r* = −0.52, *R*^2^ = 0.27). In contrast, craniosynostosis in the form of coronal suture fusion had a moderately positive Spearman correlation with serum FGF23 levels (*r* = 0.47). Notably, only those TNAP null mice with high FGF23 levels had coronal suture fusion (suture score of 2). In addition, strong positive Pearson correlations with strong correlations of determination were found for serum FGF23 levels and size normalized cranial height (*r* = 0.91, *R*^2^ = 0.82), cranial width (*r* = 0.87, *R*^2^ = 0.76) and inner canthal distance (*r* = 0.92, *R*^2^ = 0.84).

## DISCUSSION

TNAP (Tissue Non-specific Alkaline Phosphatase) enzyme deficiency leads to the metabolic disorder hypophosphatasia in mice and humans with a primary phenotype of bone hypomineralization (9, 29–31). The phenotype of severe hypophosphatasia in mouse pups and human infants can also include craniosynostosis, the premature fusion of cranial bones leading to diminished skull growth, high intracranial pressure and an abnormal skull shape (29, 38, 49). Treatment of infants and children with severe hypophosphatasia using a recombinant, mineral-targeted form of TNAP has been highly successful for improving survival, and for rescuing bone mineralization plus motor and cognitive deficiencies. Unfortunately, the recombinant enzyme therapy has been less efficacious for preventing craniosynostosis (36). In this study we primarily sought to determine if ablation of the pyrophosphate generating enzyme, ENPP1, in TNAP null mice would rescue craniosynostosis and associated craniofacial skeletal abnormalities because deletion of ENPP1 in TNAP null mice was previously shown to rescue skeletal mineralization (13) and to further understand mechanisms that mediate the craniofacial abnormalities in hypophosphatasia.

We found that body weight and body length are increased by ablation of ENPP1 in TNAP null mice, but not to the levels seen in wild type mice. This data indicates that TNAP deficiency may have impacts on body size and weight that are both dependent and independent from the TNAP-ENPP1 enzyme axis. It is worth noting that the 7-year follow up study on TNAP enzyme replacement in severely affected patients revealed that, despite enzyme replacement, the hypophosphatasia patients remained well below normal for both height and weight (36). TNAP enzyme replacement targets to mineralized tissues. It remains unknown if a TNAP function in non-mineralized tissues is responsible for the diminished body size seen in TNAP deficient mice and patients but recent studies indicate that TNAP does play a role in cell and body metabolism including brown fat thermogenesis (4, 41, 50).

Cranial height and width skull shape abnormalities of TNAP null mice were normalized to a large degree and to similar extents upon either hemi- or homozygous deletion of ENPP1. In contrast, the incidence of craniosynostosis was significantly diminished only upon homozygous deletion of ENPP1 in TNAP null mice. Cranial bone mineral density and volume fraction were also only normalized only upon homozygous and not hemizygous deletion of ENPP1 in TNAP null mice. This data is consistent to the previously published data showing rescue of skull mineralization upon genetic ablation of ENPP1 in TNAP null mice, although quantification using multiple mice was not performed at that time (13). Together our data indicates that partial normalization of the TNAP-ENPP1 enzyme balance allowing for some expression of ENPP1 (and ENPP1 generated pyrophosphate) is adequate for normalizing craniofacial skeletal shape defects, and that the abnormalities in skull shape are not dependent upon either coronal suture fusion or cranial bone mineralization. Finally, frontal and parietal bone lengths that were increased in the TNAP null mice were also increased in TNAP^−/−^/ENPP1^−/−^ mice, indicating that ENPP1 and/or ENPP1 generated PP_i_ does not mediate the increased cranial bone lengths seen in these mice. Craniosynostosis has been proposed to occur downstream of changes in frontal and/or parietal bone growth (51). Because deletion of ENPP1 does rescue coronal suture fusion but does not alter lengths of frontal and parietal bones in TNAP null mice, this indicates that increased growth of cranial bones does not likely mediate coronal suture fusion seen in the TNAP^−/−^ mice.

We also quantified trabecular and cortical bone abnormalities in long bones of the single and double mutant mice. ENPP1 null mice were previously reported to exhibit diminished femur trabecular bone volume, thickness and number by 6 weeks of age and cortical bone density, thickness and area by 22 weeks of age (27). We did not see changes in tibial trabecular or cortical bone in ENPP1 null mice which can be explained by the fact that we assessed the mice at day 17. While the differences could correspond to difference between femurs and tibias, it seems more likely that the bone abnormalities in ENPP1 null mice occur and exacerbate over time.

As stated above, genetic homozygous deletion of ENPP1 in TNAP null mice was initially reported to rescue skull, vertebral and digit hypomineralization of the TNAP^−/−^ mice (13). Subsequently, it was reported that TNAP^−/−^/ENPP1^−/−^ mice exhibited a stronger increase in mineralization of calvaria and vertebra than in digits and tibias by alizarin red staining, and micro-CT of tibias showed no difference between TNAP^−/−^/ENPP1^+/+^ and TNAP^−/−^/ENPP1^−/−^ (52). Here, upon sampling of a large number of mice, we show that deletion of ENPP1 in TNAP null mice does impact tibial bones of the mice, although it is not a clear “rescue”. TNAP^−/−^/ENPP1^+/+^ mice exhibit diminished trabecular thickness and mineral density with increased trabecular number and no difference in bone volume fraction compared to wild type mice. In contrast TNAP^−/−^/ENPP1^−/−^ mice showed decreased trabecular thickness, number and bone volume fraction plus increased trabecular spacing compared to wild type mice. Overall, deletion of ENPP1 in TNAP null mice alters but does not normalize the tibial trabecular bone abnormalities in these mice. In contrast to the tibial trabecular bone data, all measured cortical bone parameters were diminished in TNAP^−/−^ as compared to wild type mice and all of these abnormalities were normalized upon complete deletion of ENPP1.

Primary cell studies were performed to confirm that lack of TNAP and/or ENPP1 activity led to expected differences in inorganic phosphate and pyrophosphate production by the cells, and to see if previously established cellular defects in proliferation and cell metabolism of TNAP null cells were rescued upon ENPP1 ablation. Both TNAP and ENPP1 activity were significantly diminished in corresponding TNAP and/or ENPP1 null cells, confirming loss of enzyme activity upon gene deletion. As anticipated, pyrophosphate media levels were high in TNAP^−/−^/ENPP1^+/+^ cells and low in TNAP^+/+^/ENPP1^−/−^ cells when compared to that of wild type cells. Phosphate levels in media were diminished to a small but significant degree in all cells that lacked ENPP1 when other genotypes, indicating that ENPP1 is essential for generation of both phosphate and pyrophosphate when in the presence of TNAP or other pyrophosphatases. Phosphate levels in media were not diminished in TNAP null compared to wild type cells, though this may have been due to variation in the data. Consistent with our previously published results (38, 41, 46), we also found that cellular proliferation was decreased and cell metabolism was increased in TNAP^−/−^/ENPP1^+/+^ cells. Cell proliferation was not increased in TNAP^−/−^/ENPP1^−/−^ compared to TNAP^−/−^/ENPP1^+/+^ cells. This data indicates that TNAP promotes cell proliferation *via* a mechanism that is independent of ENPP1 and associated pyrophosphate. Deletion of ENPP1 only partially rescued the high cell metabolism seen in TNAP^−/−^/ENPP1^+/+^ cells, indicating that TNAP deficiency increases cell metabolic activity in part *via* ENPP1 or that TNAP and ENPP1 have independent influences on cell metabolic activity.

Overall, study of these mice revealed considerable individual variation in phenotype expression in the TNAP null mice. As stated above, Because FGF23 levels are high in ENPP1 null mice (27), because FGF23 was previously shown to suppress TNAP expression (47, 48), and because FGF23 regulates skeletal mineralization (53–55), we measured circulating levels of intact FGF23 in the mice. As anticipated based upon previously published data (27), serum FGF23 levels were significantly higher in TNAP^+/+^/ENPP1^−/−^ than in wild type mice. Serum FGF23 levels were highly variable in the TNAP^−/−^/ENPP1^+/+^ mice, with a subset of the mice at day 17 showing markedly high serum FGF23. Hemi- or homozygous deletion of ENPP1 lowered serum FGF23 levels in TNAP null mice (data not shown), indicating that high inorganic pyrophosphate levels may mediate the rise in FGF23. Because TNAP^−/−^ mice exhibit a phenotype of severe hypophosphatasia by day 17, these levels could have been caused in some manner by the phenotype itself. Therefore, we next assayed FGF23 levels in day 5 mouse serum. While results of all mouse genotypes were more variable, likely due to the inability to withdraw food prior to assay in these young mice, we again found significantly increased circulating levels of intact FGF23. In addition, we found strong to moderately strong negative correlation between serum FGF23 levels and body size, and between serum FGF23 levels and cranial bone micro-CT parameters. We also found strong positive correlation between serum FGF23 levels and craniosynostosis plus skull shape abnormalities. In other words, high levels of FGF23 correlated with poor skull bone, decreased body size, incidence of craniosynostosis and skull shape defects. While high FGF23 levels are not evident in non-lethal pediatric hypophosphatasia patients (56) and in fact low levels of the phosphatonin FGF7 were reported in this population, the TNAP^−/−^ mouse model of infantile hypophosphatasia exhibits a very severe and lethal hypophosphatasia phenotype. While entirely unanticipated, our data does demonstrate variably high serum intact FGF23 levels in this murine model of severe infantile hypophosphatasia and that high serum FGF23 correlates with severity of the phenotype. Questions remain in regards to if the FGF23 is a cause, consequence or simply a marker of phenotype severity in the TNAP^−/−^ mice, and if FGF23 may also be relevant to lethal perinatal and/or infantile human hypophosphatasia.

## Supplementary Material

Supplemental Figure 1. Sample numbers **Supplemental Figure 1 |** Sample numbers for experiments.

Supplemental Figure 2. Skull landmarks and distances **Supplemental Figure 2 |** Skull landmarks and measured distances.

## Data Availability

The raw data supporting the conclusions of this article will be made available by the authors, without undue reservation.
