# Supplementary figures and images for: Deletion of the Pyrophosphate Generating Enzyme ENPP1 Rescues Craniofacial Abnormalities in the TNAP−/− Mouse Model of Hypophosphatasia and Reveals FGF23 as a Marker of Phenotype Severity
Source: Front Dent Med. Author manuscript; Available in PMC 2022 Jul 29. (PMC9336114; doi:10.3389/fdmed.2022.846962)

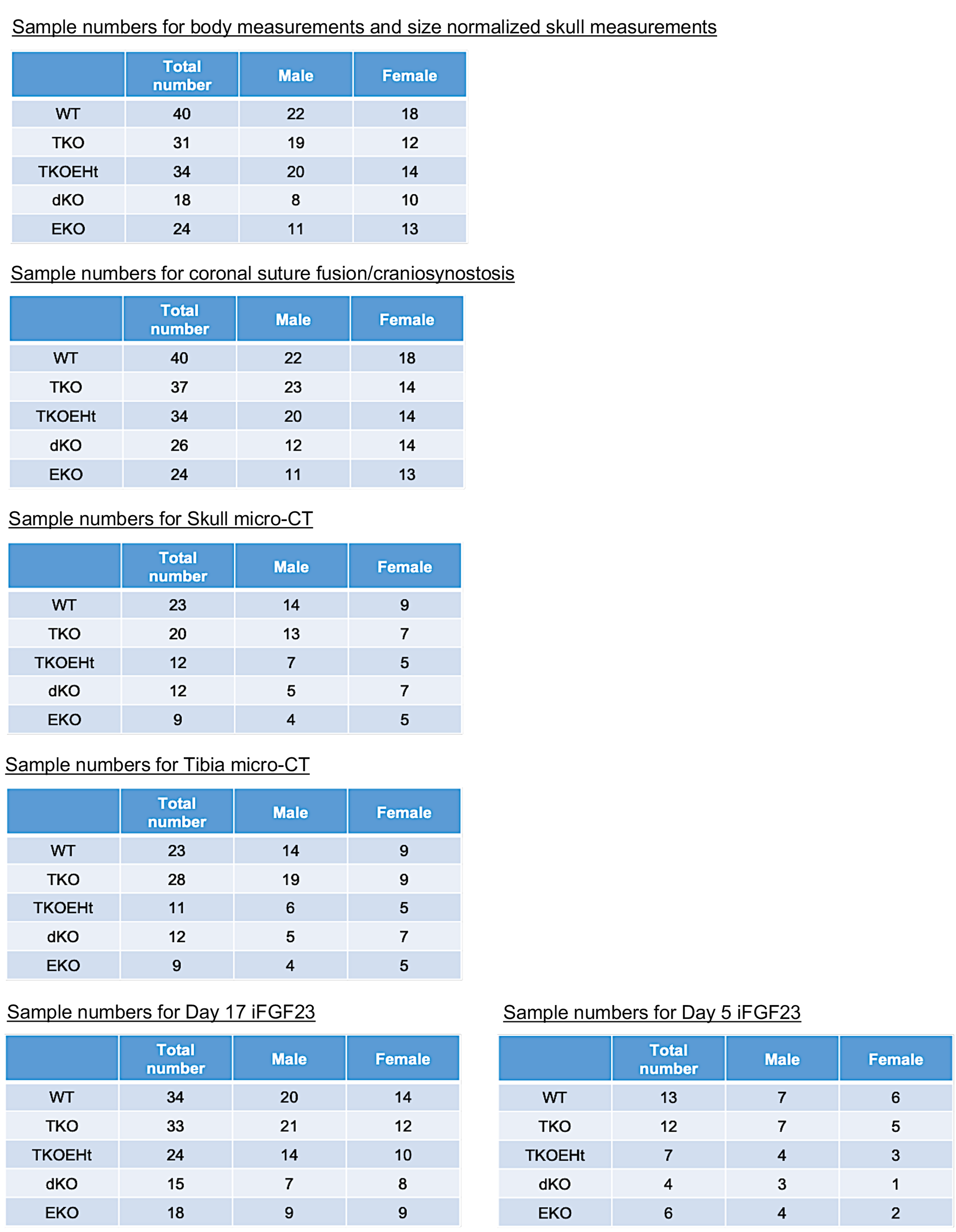

Supplement: Supplemental Figure 1. Sample numbers — Supplemental Figure 1 | Sample numbers for experiments. [file NIHMS1823833-supplement-Supplemental_Figure_1__Sample_numbers.tif]

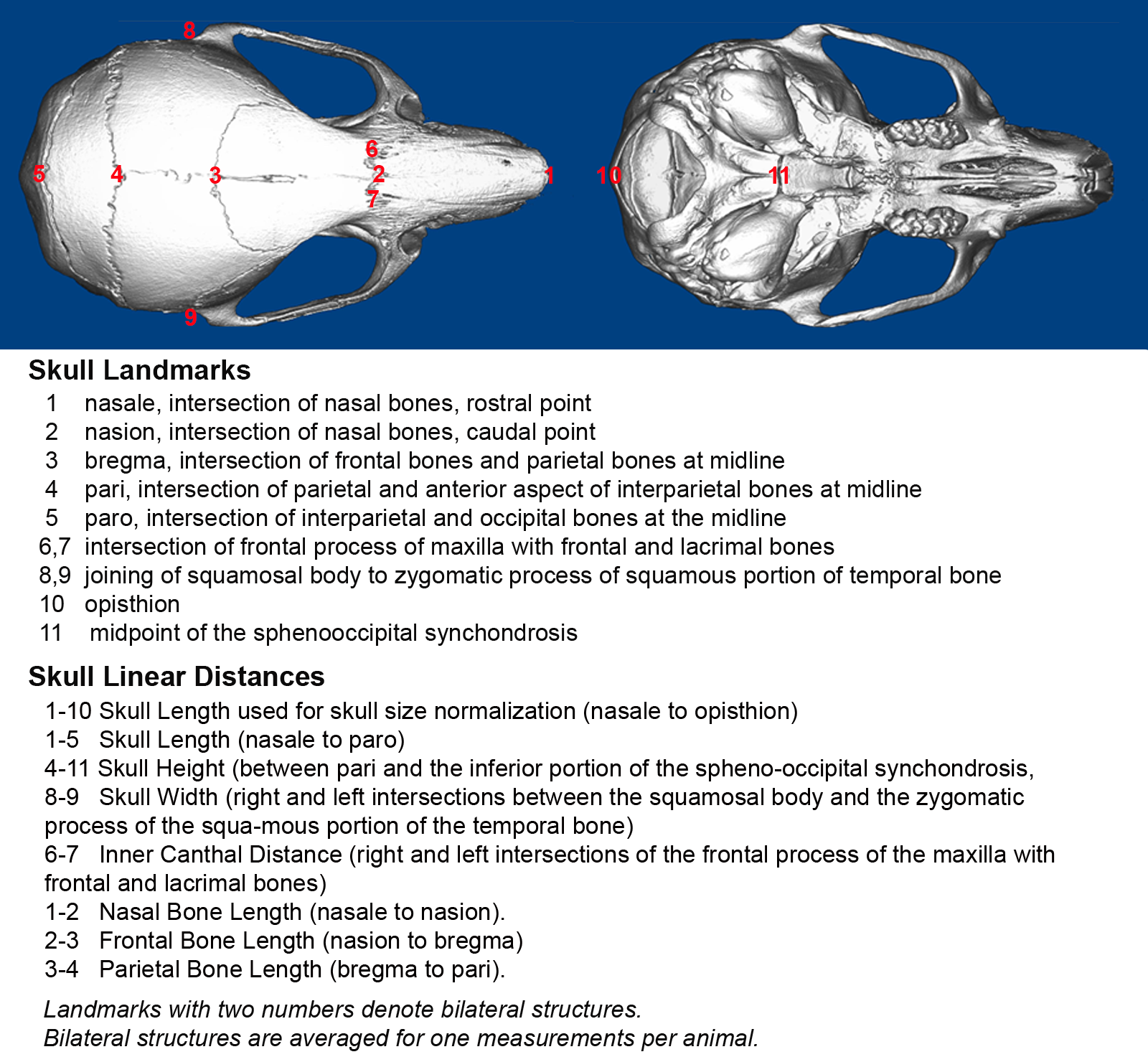

Supplement: Supplemental Figure 2. Skull landmarks and distances — Supplemental Figure 2 | Skull landmarks and measured distances. [file NIHMS1823833-supplement-Supplemental_Figure_2__Skull_landmarks_and_distances.tif]
